# Metabolic and Bariatric Surgery Utilization in the Era of Glucagon-Like Peptide-1 Receptor Agonists among Adolescents versus Adults

**DOI:** 10.1016/j.jpeds.2025.114564

**Published:** 2025-03-23

**Authors:** Sarah E. Messiah, Deepali K. Ernest, Folefac D. Atem, Faisal G. Qureshi, M. Sunil Mathew, Jackson M. Francis, Alicia Wheelington, Marlyn A. Allicock, Bethany R. Cartwright, Benjamin E. Schneider, Nestor de la Cruz-Muñoz, Steven E. Lipshultz, Ildiko Lingvay, Jaime P. Almandoz, Sarah E. Barlow

**Affiliations:** 1 Peter O’Donnell Jr. School of Public Health, University of Texas Southwestern Medical Center, Dallas, TX; 2 Department of Pediatrics, University of Texas Southwestern Medical Center, Dallas, TX; 3 Children’s Health System of Texas, Children’s and UTSW, Dallas, TX; 4 University of Texas Health Science Center at Houston, School of Public Health, Houston, TX; 5 Department of Surgery, University of Texas Southwestern Medical Center, Dallas, TX; 6 Department of Psychiatry, University of Texas Southwestern Medical Center, Dallas, TX; 7 Touchstone Diabetes Center, University of Texas Southwestern Medical Center, Dallas, TX; 8 Dewitt Daughtry Family Department of Surgery, University of Miami Miller School of Medicine, Miami, FL; 9 Department of Pediatrics, University at Buffalo Jacobs School of Medicine and Biomedical Sciences, Buffalo, NY; 10 Division of Endocrinology, Department of Internal Medicine, University of Texas Southwestern Medical Center, Dallas, TX

## Abstract

This cross-sectional study analyzed adolescent metabolic and bariatric surgery (MBS) utilization before and after glucagon-like peptide-1 receptor agonist (GLP-1RA) medication approval, comparing trends across race and ethnic groups. Although both MBS and GLP-1RA are effective weight management strategies, adolescent MBS utilization increased, while adult rates declined from 2021 through 2023.

In children and adolescents, severe obesity encompasses both class II and class III obesity.^1,2^ Severe obesity in adolescents is associated with an increased risk of cardiometabolic, hepatic, and renal diseases, impaired sleep quality, mental health challenges, greater stigma and discrimination, as well as reduced educational and professional opportunities, all of which contribute to a significantly diminished quality of life.^3^ Hispanic and non-Hispanic Black adolescents are disproportionately affected by severe obesity and associated health-related risk factors compared with their non-Hispanic White counterparts.^4^ These disparities likely reflect broader systemic, social, and structural determinants of health and underscore the importance of examining race and ethnicity as critical factors in understanding access to, and utilization of, obesity treatments.^5^

Metabolic and bariatric surgery (MBS) and glucagon-like peptide-1 receptor agonist (GLP-1RAs) medications are 2 widely used approaches for managing severe obesity and its related metabolic conditions. While MBS has been the gold standard for sustained weight loss and cardiometabolic improvements, the advent of GLP-1RAs has provided a less invasive alternative with promising results.^6^ MBS is a safe and efficacious treatment for severe obesity in adolescents,^7^ providing well-documented long-term health benefits.^8–10^ The American Academy of Pediatrics (AAP) endorses MBS for treatment of severe obesity in adolescents ≥13 years with severe obesity with qualifying comorbidities.^3^ In 2019, an AAP policy statement highlighted the need for better access to MBS for adolescents when medically indicated.^7^ Second-generation antiobesity medications, such as GLP-1RAs, achieve clinically important weight loss of 15%–20% of baseline body weight. In December 2022, semaglutide—a second-generation GLP-1RA medication—was approved to treat obesity in adolescents,^11^ lagging behind its approval for adults in June 2021. Subsequently, the AAP published its Clinical Practice Guidelines, which promoted the use of MBS and obesity medications for weight management.^12^ Given the rising prevalence of severe obesity, particularly among Hispanic and non-Hispanic Black adolescents, it is critical to assess whether these developments have influenced MBS utilization.

To understand better these trends, we analyzed MBS utilization among adolescents during the year preceding, the year of, and the year following the approval of second-generation GLP-1Ras for obesity treatment. We further compare MBS utilization between the 2 age groups by race and ethnicity. Examining MBS utilization before and after the approval of second-generation GLP-1RA medications for the treatment of obesity among adolescents will help clarify shifts in treatment patterns from 2021 to 2023 and inform strategies to improve care for adolescents with severe obesity, particularly in underserved populations.

## Methods

Data from 2021, 2022, and 2023 Metabolic and Bariatric Surgery Accreditation and Quality Improvement Program (MBSAQIP) (asmbs.org/about/mbsaqip) participant use files were analyzed. In 2012, the American College of Surgeons and the American Society for Metabolic and Bariatric Surgery merged their MBS accredited programs into the MBSAQIP.^13^ The MBSAQIP participant use file is a clinical data set of MBS patients who received their clinical care at an accredited center. Reportedly, 902 and 961 MBSAQIP participating centers were included in 2021 and 2022–2023, respectively. The University of Texas Southwestern Medical Center Institutional Review Board deemed this cohort study exempt from review and informed consent because it is a retrospective analysis of public, anonymized data sets. This study followed the Strengthening the Reporting of Observational Studies in Epidemiology reporting guideline.

*χ*^2^ tests compared preoperative descriptive characteristics by MBS completion year for adolescents. A Cochran-Armitage trend test was used to compare MBS use among adults vs adolescents from 2021 to 2023. A Cochran-Mantel-Haenszel trend test compared MBS use among adults and adolescents by race and ethnicity groups (Hispanic, non-Hispanic Black, non-Hispanic White, or other/unknown) from 2021 to 2023. Statistical analyses were performed using SAS, version 9.4 (SAS Institute, Inc) and R studio. Two-sided *P* ≤ .05 values were considered significant.

## Results

The mean age of adolescent MBS completers decreased slightly from 2021 to 2023 (17.91 years in 2021 to 17.79 years in 2023, *P* = .02). Most had a preoperative body mass index (BMI) >40 kg/m^2^, with almost 40% having a BMI >50 kg/m^2^, across all 3 study years. Laparoscopic sleeve gastrectomy remained the predominant procedure type (>86% each year). A higher prevalence of hyperlipidemia (4.0% in 2021 to 4.6% in 2023, *P* = .03) and sleep apnea (18.5% in 2021 to 23.1% in 2023, *P* < .01) were observed while the prevalence of preoperative gastroesophageal reflux disease decreased significantly (11.7% in 2021 to 8.9% in 2023, *P* = .03) (Table).

**Figure 1.**
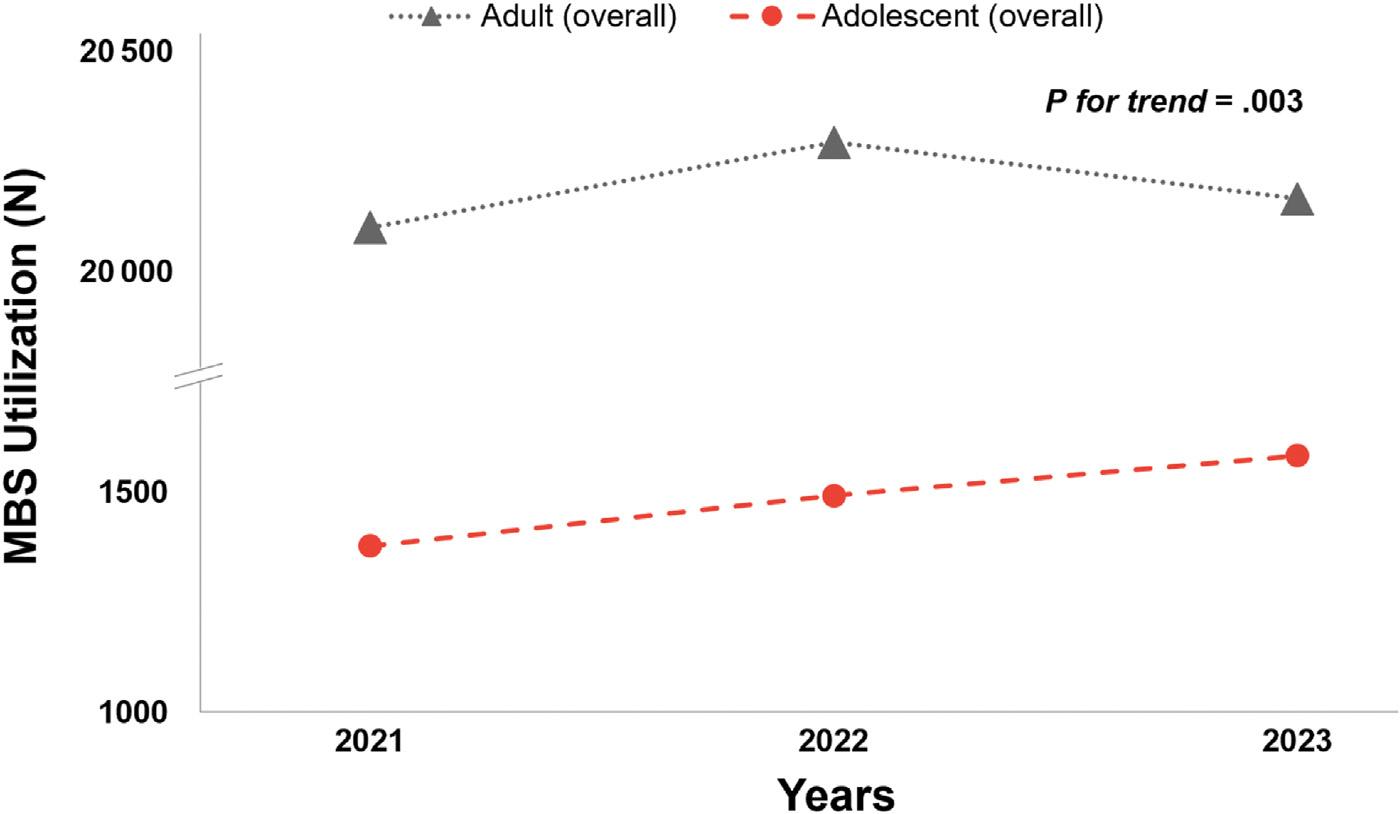
Frequency of MBS utilization among adults (≥20 years) and adolescents (13–19 years) from 2021 to 2023 (n = 4447). Trends in frequency of MBS completion from 2021 to 2023, among adults (*gray line*) and adolescents (*red line*). The adult group experienced an increase in MBS completion from 2021 (n = 209 829) to 2022 (n = 229 159). However, there was a subsequent decline in MBS completion from 2022 to 2023 (n = 216 323). The adolescent group experienced a consistent linear increase in the frequency of MBS completion from 2021 (n = 1376) to 2022 (n = 1490) to 2023 (n = 1581). Overall, MBS utilization significantly differed between adults and adolescents from 2021 to 2023 (*P* for trend = .003), with adults having consistently higher MBS utilization than adolescents.

**Figure 2.**
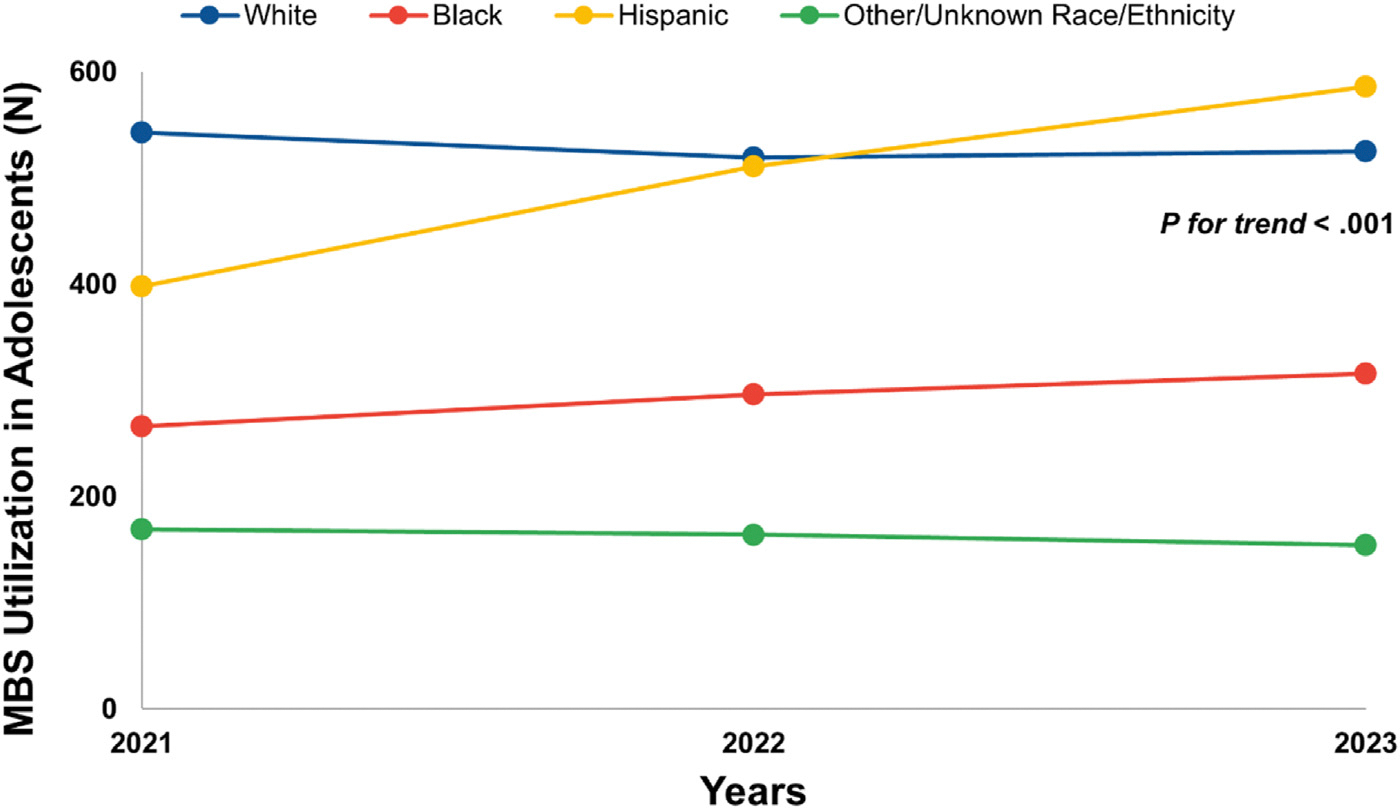
Frequency of MBS utilization in adolescents by race and ethnicity groups. Note: The other/unknown race and ethnicity group includes adolescents who identified as non-Hispanic Asian, non-Hispanic American India/Native, non-Hispanic Native Hawaiian or Pacific Islander, non-Hispanic mixed race, or other unknown race. The frequency of MBS utilization in adolescents, from 2021 to 2023, by self-reported race and ethnicity. Briefly, there was an increase in MBS utilization among non-Hispanic Black adolescents from 2021 (n = 266) to 2022 (n = 296) to (n = 316). A similar increase was observed among Hispanic adolescents from 2021 (n = 398) to 2022 (n = 511) to 2023 (n = 586). Those who identified as non-Hispanic White or other/unknown race and ethnicity had a decline in MBS completion from 2021 to 2023. Overall, MBS utilization among adolescents significantly differed by race/ethnicity groups from 2021 to 2023 (*P* for trend < .001).

**Figure 3.**
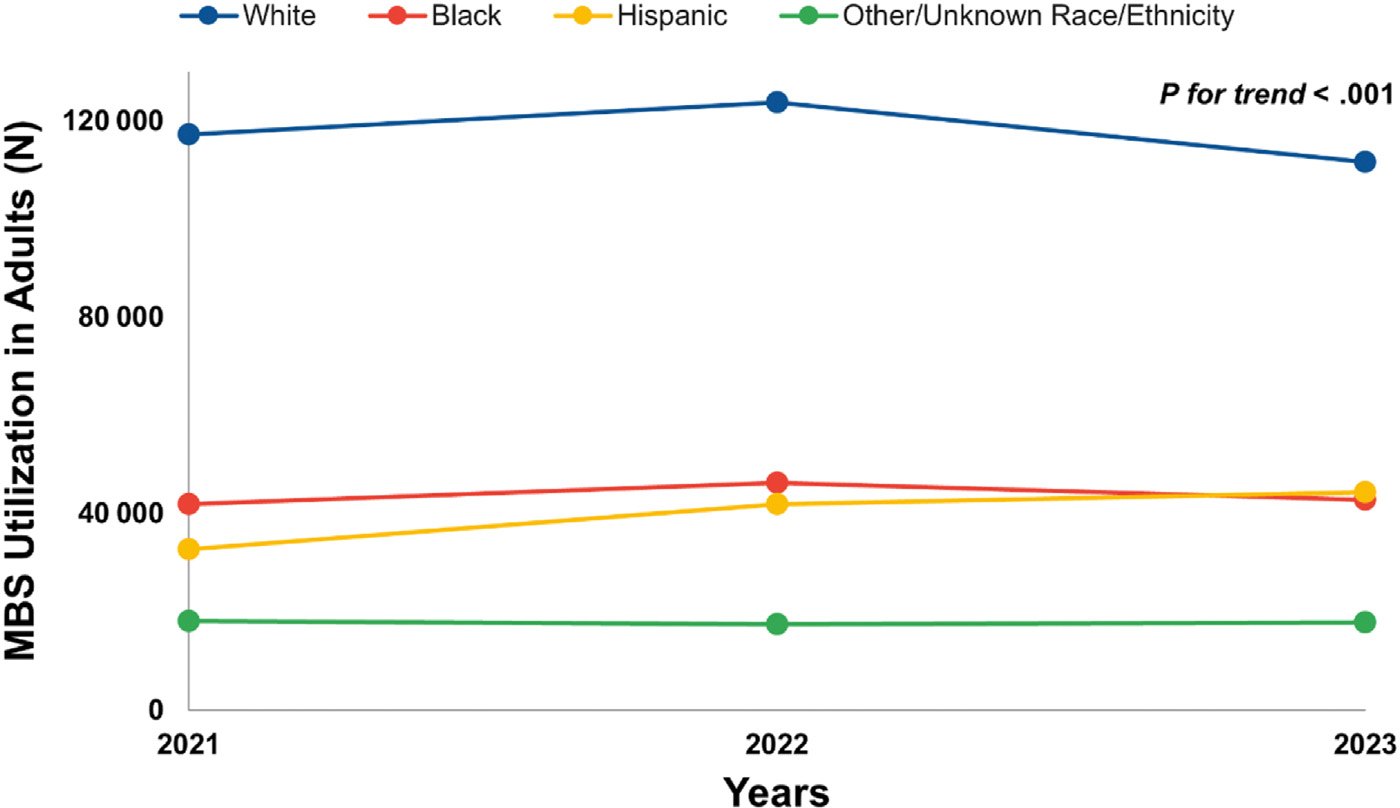
Frequency of MBS utilization in adults by race and ethnicity groups. Note: The other/unknown race and ethnicity group includes adults who identified as non-Hispanic Asian, non-Hispanic American India/Native, non-Hispanic Native Hawaiian or Pacific Islander, non-Hispanic mixed race, or other unknown race. The frequency of MBS utilization in adults, from 2021 to 2023, by self-reported race and ethnicity. Briefly, there was an increase in MBS utilization among non-Hispanic Black adults from 2021 (n = 41 851) to 2023 (n = 42 697) and Hispanic adults from 2021 (n = 32 668) to 2023 (n = 44 299). However, there was a decline in MBS utilization among non-Hispanic White adults from 2021 (n = 117 203) to 2023 (111 567), and other/unknown race from 2021 (n = 18 087) to 2023 (n = 17 760). Overall, MBS utilization among adults significantly differed by race/ethnicity groups from 2021 to 2023 (*P* for trend < .001).

A comparison of MBS utilization among adolescents vs adults demonstrated an increase among adolescents from 2021 (n = 1376) to 2022 (n = 1490) to 2023 (n = 1581), and an increase among adults from 2021 (n = 209 829) to 2022 (n = 229 159), but a decrease from 2022 to 2023 (n = 216 323) (P for trend = .003) (Figure 1). The MBS completion in adolescents increased from 2021 to 2022 to 2023 for most race/ethnicity groups (P for trend < .001 for all (Figure 2). Specifically, from 2021 to 2023, MBS completion increased from 266 to 296 to 316 procedures among non-Hispanic Black adolescents, and from 398 to 511 to 586 procedures among Hispanic adolescents. Overall, MBS utilization among adults significantly differed by race/ethnicity groups from 2021 to 2023 (P for trend <.001 for all) (Figure 3). Specifically, there was an increase in MBS utilization among non-Hispanic Black adults from 2021 (n = 41 851) to 2023 (n = 42 697) and Hispanic adults from 2021 (n = 32 668) to 2023 (n = 44 299). However, there was a decline in MBS utilization among non-Hispanic White adults from 2021 (n = 117 203) to 2023 (n = 111 567), and the other/unknown group from 2021 (n = 18 087) to 2023 (n = 17 760).

## Discussion

Analysis showed that despite the approval of second-generation GLP-1RA medications for treating obesity in adolescents in 2022, MBS utilization among US adolescents increased by almost 15% from 2021 to 2023. This finding is largely driven by increased MBS utilization among non-Hispanic Black and Hispanic adolescents, who for the first time exhibit the highest prevalence of MBS completion compared with all other racial and ethnic groups. This overall increase is consistent with our previous findings that showed an increase in MBS utilization after the release of an AAP statement advocating for improved access to surgery for adolescents.^14^

The observed increase in MBS utilization among adolescents, particularly among historically underserved populations is striking. This shift may be attributed to several factors. First, the publication of the AAP Clinical Practice Guidelines in 2023, which endorsed MBS as a safe and effective treatment for severe obesity in adolescents, likely played a role in raising awareness and improving access.^12^ In addition, systemic barriers, such as limited Medicaid coverage for antiobesity medications and widespread GLP-1RA supply shortages,^13^ may have inadvertently steered patients and providers toward MBS as a more accessible and reliable treatment option. A recent 2022–2023 analysis of privately insured adult patients showed a 2-fold increase in the use of GLP-1RA obesity medications and a 25.6% decrease in MBS utilization during the same period.^15^ The 5.6% decrease in MBS utilization from 2022 to 2023 among adults may reflect the preference for GLP-1RA medications. Another recent analysis of the 2020–2023 IQVIA Longitudinal Prescription Database showed that GLP-1RA dispensing among adolescents and young adults (up to 25 years of age) increased six-fold.^16^ However, like adults,^17^ the future of adolescent obesity treatment is likely to involve a combination of highly effective obesity medications and MBS, in addition to robust lifestyle support. This integrated approach will provide a multifaceted solution to address the rising prevalence of severe obesity, offering both pharmacological and surgical options to improve weight loss outcomes and long-term health.

This study has limitations. First, it utilizes a cross-sectional design and thus causality cannot be inferred. Second, the MBSAQIP data do not include health insurance data, and third, the MBSAQIP data set may not represent fully all MBS practices across the US. However, the analysis provides valuable insights into the rapidly evolving landscape of adolescent obesity treatment, particularly considering the recent approval of second-generation GLP-1RA obesity medications and highlights critical areas for improving access to care for underserved populations.

This study underscores the modest but increased utilization of MBS among US adolescents despite the approval and growing popularity of second-generation GLP-1RA obesity medications. This increase in adolescent MBS utilization reflects improved access and growing acceptance of MBS among providers and patients, particularly within Hispanic and Black populations. However, overall MBS utilization remains low, despite the rising national prevalence of severe obesity. Notably, nearly 40% of adolescents do not complete surgery until their BMI approaches 50 kg/m^2^. A comprehensive approach that integrates these treatments may not only improve initial weight loss but also support long-term health benefits, ultimately enhancing quality of life for adolescents with severe obesity.

## Figures and Tables

**Table.** Preoperative characteristics of adolescents (13–19 y) who completed metabolic and bariatric surgery, MBSAQIP, 2021, 2022, and 2023*

Characteristic	2021 (n = 1376)	2022 (n = 1490)	2023 (n = 1581)	*P* value

Age, mean (SD)^†^	17.91 (1.34)	17.90 (1.33)	17.79 (1.37)	**.02**
Sex, female, n (%)	1002 (72.8)	1040 (69.8)	1161 (73.4)	.15
Ethnicity and race, n (%)				**<.01**
Hispanic	398 (28.9)	511 (34.3)	586 (37.1)	
Non-Hispanic Black	266 (19.3)	296 (19.9)	316 (20.0)	
Non-Hispanic White	543 (39.5)	519 (34.8)	525 (33.2)	
Other/unknown	169 (12.3)	164 (11.0)	154 (9.7)	
Highest preoperative BMI, mean (SD)^†^	49.0 (8.2)	49.2 (8.6)	48.9 (7.9)	.70
BMI <35, n (%)	9 (0.7)	11 (0.7)	17 (1.1)	
35 < BMI < 40, n (%)	105 (7.6)	134 (9.0)	128 (8.1)	
40 < BMI < 50, n (%)	741 (53.9)	779 (52.3)	840 (53.1)	
BMI > 50, n (%)	520 (37.8)	566 (38.0)	596 (37.7)	
Preoperative BMI closest to MBS, mean (SD)^‡^	47.5 (8.1)	47.4 (8.3)	47.1 (7.7)	.54
BMI <35, n (%)	30 (2.2)	25 (1.7)	42 (2.7)	
35 ≤ BMI < 40, n (%)	174 (12.7)	212 (14.2)	215 (13.6)	
40 ≤ BMI < 50, n (%)	749 (54.5)	795 (53.4)	849 (53.7)	
BMI ≥50, n (%)	422 (30.7)	458 (30.7)	475 (30.0)	
Procedure type, n (%)				.66
LSG	1186 (86.2)	1310 (87.9)	1374 (86.9)	
LRYGB	149 (10.8)	139 (9.3)	166 (10.5)	
Other	41 (3.0)	41 (2.8)	41 (2.6)	
Preoperative comorbidities, n (%)				
Hypertension	106 (7.7)	131 (8.8)	117 (7.4)	.33
Hyperlipidemia	55 (4.0)	41 (2.8)	72 (4.6)	**.03**
Type 2 diabetes	214 (15.6)	209 (14.0)	240 (15.2)	.15
GERD	161 (11.7)	166 (11.1)	141 (8.9)	**.03**
Sleep apnea	254 (18.5)	260 (17.4)	365 (23.1)	**<.01**

*GERD*, gastroesophageal reflux disease; *LSG*, laparoscopic sleeve gastrectomy; *LRYGB*, laparoscopic Roux-en-Y gastric bypass.

Bolded *P* values indicate statistical significance at type I error (*α*) level of .05.

* Total MBSAQIP centers by year; 2021 (n = 902); 2022 (n = 961); 2023 (n = 996).

† Total missing = 51.

‡ Total missing = 1.

## Data Availability

Data sharing statement available at www.jpeds.com.

## Abbreviations

AAP: American Academy of Pediatrics
BMI: Body mass index
GLP-1RA: Glucagon-like peptide-1 receptor agonist
MBS: Metabolic and bariatric surgery
MBSAQIP: Metabolic and Bariatric Surgery Accreditation and Quality Improvement Program
US: United States
